# Supplementary material for: Sexual and psychological health of couples with azoospermia in the context of the COVID-19 pandemic
Source: PeerJ. 2021 Oct 20;9:e12162. doi: 10.7717/peerj.12162 (PMC8541304; doi:10.7717/peerj.12162)
Supplement: Supplemental Information 2 [file peerj-09-12162-s002.doc]

**Sexual Health and Psychological Health Survey Questionnaire during COVID-19 (for male)**

(This questionnaire is anonymous and confidential, please feel free to fill it out.)

We are very grateful for your contributions to this research, and wish you all the best and realize your dreams soon.

Age: Height: cm Weight: kg

What type of profession do you do?

□Civil servant □Professional and technical personnel □Business management personnel □Staff □Worker □Farmer □Self-employed □None □Others: ____________

Personal annual income □<50,000 yuan □50,000-100,000 yuan □100,000-150,000 yuan □150,000-200,000 yuan □>200,000 yuan

How many years have you planed to have a child? : _________ years

Entertainment: ___________

Do you take drugs? □Yes, what kind of drugs:___________ □No

Time spent on cell phones per day: ___________ hours

Sexual life frequency: ___________ times per month

Education level □high school and below □college for professional training □undergraduate □postgraduate and above

**For the following questions, please choose a check mark before the choice that you think best suits your situation during COVID-19 pandemic.**

Diet □ only vegetarian food □ mainly meat □ half vegetarian food and half vegetarian meat

Is there a big stress in work and life? □very high □high □general □low □no

Frequency of physical exercise □None □2 times a month or less

□1 time a week □2 times a week and above

Smoking □Yes,______ cigarettes per day □No

Drinking □Almost every day □ Often □ Sometimes □ Rarely □ Never

Coffee □Almost every day □Always □Sometimes □Rarely □Never

1. Does the novel coronavirus pneumonia pandemic make you anxious?

□Severe □Slight □None

2. Compared with that before the COVID-19 pandemic, how is your relationship with your partner?

□Very good □Fair □Deteriorating

3. During the novel coronavirus pneumonia pandemic, how does your relationship compared to before?

□Better □No change □Worse

4. Compared with that before the COVID-19 pandemic, how has your sexual desire changed?

□Increased □Unchanged □Decreased

5. Compared with that before the COVID-19 pandemic, how has your sexual frequency changed?

□Increased □Unchanged □Decreased

6. Compared with that before the COVID-19 pandemic, how has your sexual satisfaction changed?

□Increased □Unchanged □Decreased

7. During the new coronavirus pneumonia pandemic, what about your drinking before or during sexual activity?

□Increased □Unchanged □Decreased

8. How has your frequency of masturbation changed compared with that before the COVID-19 pandemic?

□Increased □Unchanged □Decreased □ None

9. How has your frequency of pornography use changed compared with that before the COVID-19 pandemic?

□Increased □Unchanged □Decreased □ None

10. During the new coronavirus pneumonia pandemic, how does your condom use frequency (in sexual contact) change?

□Increased □Unchanged □Decreased

11.How is your annual income affected by the new coronavirus pneumonia?

□Increased □Unchanged □Decreased

12. Have you postponed your plan to have a child because of the new coronary pneumonia?

□Yes □No How long？

13. Have you encountered difficulties in receiving fertility treatment due to new coronary pneumonia?

□Yes □No

If you have, what are the difficulties?_______________________________________________

14. In the process of receiving fertility treatment, has the new coronavirus nucleic acid test increased your burden?

□Yes □No

15. Is sex important to you?

□Very important □Important □Average □Not important □Very unimportant

16. How satisfied do you feel about the relationship with your wife during your sex life?

□No sexual activity □Very satisfied □Relatively satisfied □Half of satisfaction and dissatisfaction □Dissatisfied □Very dissatisfied

17. Are you satisfied with your sex life?

□No sexual activity □Very satisfied □Relatively satisfied □Half of satisfaction and dissatisfaction □Dissatisfied □very dissatisfied

18. Have you experienced sexual intercourse difficulties?

□Never □Very rarely □Rarely □Sometimes □Always □Almost always

19. Intercourse time

□<1 minute □1-2 minutes □3-4 minutes □5-7 minutes □8-10 minutes

□11-15 minutes □16-30 minutes □>30 minutes

20. Foreplay time

□<1 minute □2-10 minutes □11-20 minutes □21-30 minutes □31-60 minutes

□>60 minutes

21. The possibility of masturbating to get an orgasm.

□Almost always □Always □Sometimes □Rarely □Never □Never tried

22. The possibility of orgasm through non-coital intercourse (such as foreplay or oral sex).

□Almost always □Always □Sometimes □Rarely □Never □never tried

23. The possibility of orgasm in sexual intercourse.

□Almost always □Always □Sometimes □Rarely □Never □never tried

**THE INTERNATIONAL INDEX OF ERECTILE FUNCTION**

**(IIEF)**

1.How often were you able to get an erection during sexual

activity?

No sexual activity

□ Almost never/never

□ A few times (much less than half the time)

□ Sometimes (about half the time)

□ Most times (much more than half the time)

□ Almost always/always

2. When you had erections with sexual stimulation, how

often were your erections hard enough for penetration?

□ No sexual activity

□ Almost never/never

□A few times (much less than half the time)

□ Sometimes (about half the time)

□ Most times (much more than half the time)

□ Almost always/always

3. When you attempted sexual intercourse, how often were

you able to penetrate (enter) your partner?

□ Did not attempt intercourse

□ Almost never/never

□ A few times (much less than half the time)

□ Sometimes (about half the time)

□ Most times (much more than half the time)

□ Almost always/always

4. During sexual intercourse, how often were you able to

maintain your erection after you had penetrated (entered)

your partner?

□ Did not attempt intercourse

□ Almost never/never

□ A few times (much less than half the time)

□ Sometimes (about half the time)

□ Most times (much more than half the time)

□ Almost always/always

5. During sexual intercourse, how difficult was it to maintain

your erection to completion of intercourse?

□ Did not attempt intercourse

□ Extremely difficult

□ Very difficult

□ Difficult

□ Slightly difficult

□ Not difficult

6. How many times have you attempted sexual intercourse?

□ No attempts

□ One to two attempts

□ Three to four attempts

□ Five to six attempts

□ Seven to ten attempts

□ Eleven+ attempts

7. When you attempted sexual intercourse, how often was it

satisfactory for you?

□ Did not attempt intercourse

□ Almost never/never

□ A few times (much less than half the time)

□ Sometimes (about half the time)

□ Most times (much more than half the time)

□ Almost always/always

8. How much have you enjoyed sexual intercourse?

□ No intercourse

□ No enjoyment

□ Not very enjoyable

□ Fairly enjoyable

□ Highly enjoyable

□ Very highly enjoyable

9. When you had sexual stimulation or intercourse, how

often did you ejaculate?

□ No sexual stimulation/intercourse

□Almost never/never

□A few times (much less than half the time)

□ Sometimes (about half the time)

□ Most times (much more than half the time)

□ Almost always/always

10. When you had sexual stimulation or intercourse, how

often did you have tile feeling of orgasm or climax?

□ No sexual stimulation/intercourse

□ Almost never/never

□ A few times (much less than half the time)

□Sometimes (about half the time)

□ Most times (much more than half the time)

□ Almost always/always

11. How often have you felt sexual desire?

□ Almost never/never

□ A few times (much less than half the time)

□ Sometimes (about half the time)

□ Most times (much more than half the time)

□ Almost always/always

12. How would you rate your level of sexual desire?

□ Very low/none at all

□ Low

□ Moderate

□ High

□ Very high

13. How satisfied have you been with your overall sex life?

□ Very dissatisfied

□ Moderately dissatisfied

□About equally satisfied and dissatisfied

□ Moderately satisfied

□ Very satisfied

14. How satisfied have you been with your sexual

relationship with your partner?

□ Very dissatisfied

□ Moderately dissatisfied

□About equally satisfied and dissatisfied

□ Moderately satisfied

□ Very satisfied

15. How do you rate your confidence that you could get and

keep an erection?

□ Very low

□ Low

□ Moderate

□ High

□ Very high

**Premature Ejaculation Diagnostic Tool**

1. During sex, how difficult is it for you to try to prolong ejaculation?

□No difficulty □Some difficulty □Difficult □Very difficult □Extremely difficult

2. Is ejaculation before your will?

□Almost no/nothing (0%)

□ Less than half the frequency (25%)

□About half the frequency (50%)

□More than half the frequency (75%)

□Almost all/all (100%)

3. Ejaculate with little stimulation?

□Almost no/nothing (0%)

□ Less than half the frequency (25%)

□About half the frequency (50%)

□More than half the frequency (75%)

□Almost all/all (100%)

4. How many times do you feel anxious, nervous, etc. after ejaculation (not your own will)?

□never □some □medium □very □extremely

5. Do you pay more attention to the dissatisfaction of your sexual partner due to the time of your ejaculation?

□never □some □medium □very □extremely

**The Generalized Anxiety Disorder Screener (GAD-7)**

In the past two weeks, how often did the following symptoms appear in your life?

1.Feeling nervous, anxious or on edge

□No □A few days □More than half of the time □Almost every day

2.Not being able to stop or control worrying

□No □A few days □More than half of the time □Almost every day

3.Worrying too much about different things

□No □A few days □More than half of the time □Almost every day

4.Trouble relaxing

□No □A few days □More than half of the time □Almost every day

5.Being so restless that it is hard to sit still

□No □A few days □More than half of the time □Almost every day

6.Becoming easily annoyed or irritable

□No □A few days □More than half of the time □Almost every day

7.Feeling afraid as if something awful might happen

□No □A few days □More than half of the time □Almost every day

**The Patient Health Questionnaire-9(PHQ-9)**

In the past two weeks, how often did the following symptoms appear in your life?

1 Little interest or pleasure in doing things

□No □A few days □More than half of the time □Almost every day

2 Feeling down, depressed, or hopeless

□No □A few days □More than half of the time □Almost every day

3 Difficulty falling or staying asleep, or sleeping too much

□No □A few days □More than half of the time □Almost every day

4 Feeling tired or having little energy

□No □A few days □More than half of the time □Almost every day

5 Poor appetite or overeating

□No □A few days □More than half of the time □Almost every day

6 I am dissatisfied with myself, feel that I am a failure, or embarrass my family

□No □A few days □More than half of the time □Almost every day

7 Trouble concentrating on things, such as reading the newspaper or watching TV

□No □A few days □More than half of the time □Almost every day

8 Moving or speaking so slowly that other people could have noticed? Or the opposite—being so fidgety or restless that you have been moving around a lot more than usual.

□No □A few days □More than half of the time □Almost every day

9 Thoughts that you would be better off dead or of hurting yourself in some way

□No □A few days □More than half of the time □Almost every day

**The Quality of Marriage Index (QMI):**

Please use a 7-point scale to rate the following questions, from 1 (strongly disagree) to 7 (strongly agree)

1. We have a good marriage.

2 My relationship with my partner is very stable

3 Our marriage is strong.

4 My relationship with my partner makes me happy.

5 I really feel like part of a team with my partner.

Please use a 10-point scale to rate the overall happiness of you and your spouse, from 1 (very low) to 10 (very high).

6 The degree of happiness, everything considered, in our marriage is
