# Supplementary material for: Sexual and psychological health of couples with azoospermia in the context of the COVID-19 pandemic
Source: PeerJ. 2021 Oct 20;9:e12162. doi: 10.7717/peerj.12162 (PMC8541304; doi:10.7717/peerj.12162)
Supplement: Supplemental Information 3 [file peerj-09-12162-s003.doc]

**Sexual Health and Psychological Health Survey Questionnaire during COVID-19 (for female)**

(This questionnaire is anonymous and confidential, please feel free to fill it out.)

We are very grateful for your contributions to this research, and wish you all the best and realize your dreams soon.

Age: Height: cm Weight: kg

What type of profession do you do?

□Civil servant □Professional and technical personnel □Business management personnel □Staff □Worker □Farmer □Self-employed □None □Others: ____________

Personal annual income □<50,000 yuan □50,000-100,000 yuan □100,000-150,000 yuan □150,000-200,000 yuan □>200,000 yuan

How many years have you planed to have a child?

: _________ years Entertainment: ___________

Do you take drugs? □Yes, what kind of drugs:___________ □No

Time spent on cell phones per day: ___________ hours

Sexual life frequency: ___________ times per month

Education level □high school and below □college for professional training □undergraduate □postgraduate and above

**For the following questions, please choose a check mark before the choice that you think best suits your situation during COVID-19 pandemic.**

Diet □ only vegetarian food □ mainly meat □ half vegetarian food and half vegetarian meat

Is there a big stress in work and life? □very high □high □general □low □no

Frequency of physical exercise □None □2 times a month or less

□1 time a week □2 times a week and above

Smoking □Yes,______ cigarettes per day □No

Drinking □Almost every day □ Often □ Sometimes □ Rarely □ Never

Coffee □Almost every day □Always □Sometimes □Rarely □Never

1. Does the novel coronavirus pneumonia pandemic make you anxious?

□Severe □Slight □None

2. Compared with that before the COVID-19 pandemic, how is your relationship with your partner?

□Very good □Fair □Deteriorating

3. During the novel coronavirus pneumonia pandemic, how does your relationship compared to before?

□Better □No change □Worse

4. Compared with that before the COVID-19 pandemic, how has your sexual desire changed?

□Increased □Unchanged □Decreased

5. Compared with that before the COVID-19 pandemic, how has your sexual frequency changed?

□Increased □Unchanged □Decreased

6. Compared with that before the COVID-19 pandemic, how has your sexual satisfaction changed?

□Increased □Unchanged □Decreased

7. During the new coronavirus pneumonia pandemic, what about your drinking before or during sexual activity?

□Increased □Unchanged □Decreased

8. How has your frequency of masturbation changed compared with that before the COVID-19 pandemic?

□Increased □Unchanged □Decreased □ None

9. How has your frequency of pornography use changed compared with that before the COVID-19 pandemic?

□Increased □Unchanged □Decreased □ None

10. During the new coronavirus pneumonia pandemic, how does your condom use frequency (in sexual contact) change?

□Increased □Unchanged □Decreased

11.How is your annual income affected by the new coronavirus pneumonia?

□Increased □Unchanged □Decreased

12. Have you postponed your plan to have a child because of the new coronary pneumonia? How long？

□Yes □No

13. Have you encountered difficulties in receiving fertility treatment due to new coronary pneumonia?

□Yes □No

If you have, what are the difficulties? ________________________________________________

14. In the process of receiving fertility treatment, has the new coronavirus nucleic acid test increased your burden?

□Yes □No

15. Is sex important to you?

□Very important □Important □General □Unimportant □Very unimportant

16. Over the past 4 weeks, how often did you feel sexual desire or interest?

□Almost always or always

□Most times (more than half the time)

□Sometimes (about half the time)

□A few times (less than half the time)

□Almost never or never

17. Over the past 4 weeks, how would you rate your level (degree) of sexual desire or

interest?

□Very high □High □Moderate □Low □Very low or none at all

18.Over the past 4 weeks, how often did you feel sexually aroused (“turned on”) during sexual activity or intercourse?

□Almost always or always

□Most times (more than half the time)

□Sometimes (about half the time)

□A few times (less than half the time)

□Almost never or never

19.Over the past 4 weeks, how would you rate your level of sexual arousal (“turn on”)

during sexual activity or intercourse?

□No sexual activity

□Very high

□High

□Moderate

□Low

□Very low or none at all

20.Over the past 4 weeks, how confident were you about becoming sexually aroused

during sexual activity or intercourse?

□ No sexual activity

□ Very high confidence

□ High confidence

□ Moderate confidence

□ Low confidence

□ Very low or no confidence

21.Over the past 4 weeks, how often have you been satisfied with your arousal (excitement) during sexual activity or intercourse? Response Options

□ No sexual activity

□Almost always or always

□Most times (more than half the time)

□Sometimes (about half the time)

□A few times (less than half the time)

□Almost never or never

22.Over the past 4 weeks, how often did you become lubricated (“wet”) during sexual

activity or intercourse?

□ No sexual activity

□Almost always or always

□Most times (more than half the time)

□Sometimes (about half the time)

□A few times (less than half the time)

□Almost never or never

23.Over the past 4 weeks, how difficult was it to become lubricated (“wet”) during sexual activity or intercourse?

□ No sexual activity

□ Extremely difficult or impossible

□ Very difficult

□ Difficult

□ Slightly difficult

□ Not difficult

24.Over the past 4 weeks, how often did you maintain your lubrication (“wetness”) until

completion of sexual activity or intercourse?

□ No sexual activity

□Almost always or always

□Most times (more than half the time)

□Sometimes (about half the time)

□A few times (less than half the time)

□Almost never or never

25.Over the past 4 weeks, how difficult was it to maintain your lubrication (“wetness”)

until completion of sexual activity or intercourse?

□ No sexual activity

□ Extremely difficult or impossible

□ Very difficult

□ Difficult

□ Slightly difficult

□ Not difficult

26.Over the past 4 weeks, when you had sexual stimulation or intercourse, how often

did you reach orgasm (climax)?

□ No sexual activity

□Almost always or always

□Most times (more than half the time)

□Sometimes (about half the time)

□A few times (less than half the time)

□Almost never or never

27.Over the past 4 weeks, when you had sexual stimulation or intercourse, how diffi-

cult was it for you to reach orgasm (climax)?

□ No sexual activity

□ Extremely difficult or impossible

□ Very difficult

□ Difficult

□ Slightly difficult

□ Not difficult

28.How satisfied have you been with the amount of emotional closeness during sexual activity between you and your partner?

□ No sexual activity

□ Very satisfied

□ Moderately satisfied

□About equally satisfied and dissatisfied

□ Moderately dissatisfied

□ Very dissatisfied

29.Over the past 4 weeks, how satisfied have you been with the amount of emotional closeness during sexual activity between you and your partner?

□ No sexual activity

□ Very satisfied

□ Moderately satisfied

□About equally satisfied and dissatisfied

□ Moderately dissatisfied

□ Very dissatisfied

30.how satisfied have you been with your sexual relationship with your partner?

□ Very satisfied

□ Moderately satisfied

□About equally satisfied and dissatisfied

□ Moderately dissatisfied

□ Very dissatisfied

31.Over the past 4 weeks, how satisfied have you been with your overall sexual life?

□ Very satisfied

□ Moderately satisfied

□About equally satisfied and dissatisfied

□ Moderately dissatisfied

□ Very dissatisfied

32.Over the past 4 weeks, how often did you experience discomfort or pain during

vaginal penetration?

□ Did not attempt intercourse

□ Almost always or always

□ Most times (more than half the time)

□ Sometimes (about half the time)

□ A few times (less than half the time)

□ Almost never or never

33.Over the past 4 weeks, how often did you experience discomfort or pain following vaginal penetration?

□ Did not attempt intercourse

□ Almost always or always

□Most times (more than half the time)

□ Sometimes (about half the time)

□ A few times (less than half the time)

□ Almost never or never

34.Over the past 4 weeks, how would you rate your level (degree) of discomfort or pain

during or following vaginal penetration?

□ Did not attempt intercourse

□ Very high

□ High

□ Moderate

□ Low

□Very low or none at all

35. Intercourse time

□<1 minute □1-2 minutes □3-4 minutes □5-7 minutes □8-10 minutes

□11-15 minutes □16-30 minutes □>30 minutes

36. Foreplay time

□<1 minute □2-10 minutes □11-20 minutes □21-30 minutes □31-60 minutes

□>60 minutes

37. The possibility of reaching orgasm through masturbating.

□Almost always □Always □Sometimes □Rarely □Never □Never tried

38. The possibility of reaching orgasm in non-coital intercourse (such as foreplay or oral sex)

□Almost always □Always □Sometimes □Rarely □Never □Never tried

39. The possibility of having an orgasm during sexual intercourse

□Almost always □Always □Sometimes □Rarely □Never □Never tried

**The Generalized Anxiety Disorder Screener (GAD-7)**

In the past two weeks, how often did the following symptoms appear in your life?

1.Feeling nervous, anxious or on edge

□No □A few days □More than half of the time □Almost every day

2.Not being able to stop or control worrying

□No □A few days □More than half of the time □Almost every day

3.Worrying too much about different things

□No □A few days □More than half of the time □Almost every day

4.Trouble relaxing

□No □A few days □More than half of the time □Almost every day

5.Being so restless that it is hard to sit still

□No □A few days □More than half of the time □Almost every day

6.Becoming easily annoyed or irritable

□No □A few days □More than half of the time □Almost every day

7.Feeling afraid as if something awful might happen

□No □A few days □More than half of the time □Almost every day

**The Patient Health Questionnaire-9(PHQ-9)**

In the past two weeks, how often did the following symptoms appear in your life?

1 Little interest or pleasure in doing things

□No □A few days □More than half of the time □Almost every day

2 Feeling down, depressed, or hopeless

□No □A few days □More than half of the time □Almost every day

3 Difficulty falling or staying asleep, or sleeping too much

□No □A few days □More than half of the time □Almost every day

4 Feeling tired or having little energy

□No □A few days □More than half of the time □Almost every day

5 Poor appetite or overeating

□No □A few days □More than half of the time □Almost every day

6 I am dissatisfied with myself, feel that I am a failure, or embarrass my family

□No □A few days □More than half of the time □Almost every day

7 Trouble concentrating on things, such as reading the newspaper or watching TV

□No □A few days □More than half of the time □Almost every day

8 Moving or speaking so slowly that other people could have noticed? Or the opposite—being so fidgety or restless that you have been moving around a lot more than usual.

□No □A few days □More than half of the time □Almost every day

9 Thoughts that you would be better off dead or of hurting yourself in some way

□No □A few days □More than half of the time □Almost every day

**The Quality of Marriage Index (QMI):**

Please use a 7-point scale to rate the following questions, from 1 (strongly disagree) to 7 (strongly agree)

1. We have a good marriage.

2 My relationship with my partner is very stable

3 Our marriage is strong.

4 My relationship with my partner makes me happy.

5 I really feel like part of a team with my partner.

Please use a 10-point scale to rate the overall happiness of you and your spouse, from 1 (very low) to 10 (very high).

6 The degree of happiness, everything considered, in our marriage is
