# Supplementary material for: Sexual and psychological health of couples with azoospermia in the context of the COVID-19 pandemic
Source: PeerJ. 2021 Oct 20;9:e12162. doi: 10.7717/peerj.12162 (PMC8541304; doi:10.7717/peerj.12162)
Supplement: Supplemental Information 4 [file peerj-09-12162-s004.docx]

**性健康及心理健康调查问卷（男）**

**（此问卷是匿名及保密的，请您放心填写）**

**我们非常感谢及感激您对此项研究做出的贡献，祝您一切顺利，早日圆梦。**

年龄： 身高： cm 体重 ： kg

从事何种类型工作 □公务员 □专业技术人员 □企业管理人员 □职员

□工人 □农民 □个体户 □无 □其它：____________

个人年收入 □<5万元 □5-10万元 □10-15万元 □15-20万元 □＞20万元

要孩子几年了：_________ 年 娱乐方式：___________

是否服用药物： □是，何种药物：___________ □ 否

**每天**看手机时间： 小时

**每月**性生活次数： 次

**以下题目请您选择一个您认为最符合您的情况的打对号**

教育程度 □高中及以下 □大专 □大学本科 □研究生及以上

饮食 □只吃素食 □以肉食为主 □素食肉食一半一半

工作及生活压力大吗 □非常大 □大 □一般 □不大 □没有

体育锻炼频率 □没有 □一个月2次及以下

□一周1次 □一周2次及以上

吸烟 □是，一天 支 □ 否

饮酒 □几乎每天 □常常 □有时 □很少 □从不

咖啡 □几乎每天 □常常 □有时 □很少 □从不

1.新型冠状病毒肺炎，是否给您带来焦虑？

□是 □有一点 □ 否

2.在新型冠状病毒肺炎大流行期间，您的伴侣关系如何?

□很好 □一般 □恶化

3.在新型冠状病毒肺炎大流行期间，您的伴侣关系与之前相比如何?

□变好 □无变化 □变坏

4.在新型冠状病毒肺炎大流行期间，您的性欲与之前相比如何?

□增加 □不变 □减少

5.在新型冠状病毒肺炎大流行期间，您的性行为频率与之前相比如何?

□增加 □不变 □减少

6.在新型冠状病毒肺炎大流行期间，您的性生活满意度与之前相比如何?

□增加 □不变 □减少

7.在新型冠状病毒肺炎大流行期间，您在性活动之前或期间饮酒情况如何?

□增加 □不变 □减少

8.在新型冠状病毒肺炎流行期间，您的手淫频率是如何变化的?

□增加 □不变 □减少 □从来没有

9.在新型冠状病毒肺炎大流行期间，您们使用色情作品的频率有何变化?

□增加 □不变 □减少 □从来没有

10.在新型冠状病毒肺炎大流行期间，您的避孕套使用比例(在性接触中)有何变化?

□增加 □不变 □减少

11.新型冠状病毒肺炎，是否影响了您的年收入？

□增加 □不变 □减少

12.您是否因为新冠肺炎，想要推迟要孩子的时间

□是 □否

13.您是否因新冠肺炎的原因，在接受助孕治疗过程中遇到困难？

□是 □否

**如果遇到困难，哪方面的困难：**

14.在接受辅助生殖技术助孕过程中，进行新冠病毒核酸检测，是否增加了您的负担？

□是 □否

15.性生活对您来说重要吗？

□非常重要 □重要 □一般 □不重要 □非常不重要

16.在性生活过程中您与妻子的感情亲密度满意程度怎么样？

□没有性行为 □非常满意 □比较满意 □满意和不满各占一半

□不满意 □非常不满意

17.您对您的性生活满意吗？

□没有性行为 □非常满意 □比较满意 □满意和不满各占一半

□不满意 □非常不满意

18.您是否有性交困难，性交障碍？

□从不 □极少 □很少 □有时 □常常 □几乎总是

19.性交时间

□<1分钟 □1-2 分钟 □3-4 分钟 □5-7分钟 □8-10 分钟

□11-15 分钟 □16-30 分钟 □>30 分钟

20.前戏时间

□<1分钟 □2-10分钟 □11-20分钟 □21-30分钟 □31-60分钟

□>60分钟

21.手淫获得高潮的可能

□几乎总是 □常常 □有时 □很少 □从不 □没有尝试过

22.非性交行为（如爱抚或者口交等）获得高潮的可能

□几乎总是 □常常 □有时 □很少 □从不 □没有尝试过

23.性交行为获得高潮的可能

□几乎总是 □ 常常 □有时 □很少 □从不 □没有尝试过

1、近四周内您在性活动中有多少时候阴茎能达到勃起？

□ 几乎总是能达成或总是能达到勃起

□ 多数时候能达到勃起（远多于一半时候）

□ 有时能达到勃起（约一半时候）

□ 少数几次能达到勃起（远少于一半时候）

□ 几乎没有或没有达到勃起

2、最近四周内您因性刺激而有阴茎勃起时，有多少时候感到阴茎强度足够插入配偶的体内？

□ 没有性刺激

□ 几乎总是或总是感到硬度足够

□ 多数时候感到硬度足够（远多于一半时候）

□ 有时感到硬度足够（约一半时候）

□ 少数几次感到硬度足够（远少于一半时候）

□ 几乎没有感到或没有感到硬度足够

3、近四周内您尝试性交时阴茎有多少能够插入（进入）您配偶的体内？

□ 几乎总是能够或总是插入

□ 多数时候能够插入（远多于一半时候）

□ 有时能够插入（约一半时候）

□ 少数几次能够插入（远少于一半时候）

□ 几乎不能够或不能够插入

4、最近四周内您性交时阴茎插入（进入）配偶体内后，有多少时候能够维持勃起状态？

□ 几乎总是能够或总是能够维持勃起

□ 多数时候能够维持勃起（远多于一半时候）

□ 有时能够维持勃起（约一半时候）

□ 少数几次能够维持勃起（远少于一半时候）

□ 几乎不能够或不能够维持勃起

5、最近四周您性交时维持阴茎勃起直到性交完毕有多大困难？

□ 困难极大

□ 困难很大

□ 困难

□ 有点困难

□ 不困难

6、最近四周内您尝试性交的次数有多少？

□ 1～2 次

□ 3～4 次

□ 5～6 次

□ 7～10 次

□ 11 次以上

7、最近四周内您尝试性交时有多少时候感到满足？

□ 几乎总是感到满足或总是感到满足

□ 多数时候感到满足（远多于一半时候）

□ 有时感到满足（约一半时候）

□ 少数几次感到满足（远少于一半时候）

□ 几乎没有感到满足或没有感到满足

8、最近四周内您多大程度上享受到性交的快乐？

□ 享受到极度快乐

□ 享受到高度快乐

□ 享受到一般快乐

□ 较少享受到快乐

□ 没有享受到快乐

9、最近四周内您受到性刺激或性交时，有多少时候伴有射精？

□ 几乎总是伴有射精或总是伴有射精

□ 多数时候伴有射精（远多于一半时候）

□ 有时伴有射精（约一半时候）

□ 少数几次伴有射精（远少于一半时候）

□ 几乎不或不伴有射精

10、最近四周内您受到性刺激或性交时，有多少时候有性高潮的感觉（不论有没有射精）？

□ 几乎总是有或总是有性高潮感觉

□ 多数时候有性高潮感觉（远多于一半时候）

□ 有时有性高潮感觉（约一半时候）

□ 少数几次有性高潮感觉（远少于一半时候）

□ 几乎没有或没有性高潮感觉

11、最近四周内您多少时候感觉有性欲？

□ 几乎总是有或总是有性欲

□ 多数时候有性欲（远多于一半时候）

□ 有时有性欲（约一半时候）

□ 少数几次有性欲（远少于一半时候）

□ 几乎没有或没有性欲

12、您对最近四周内您的性欲程度如何评价？

□ 很高

□ 高

□ 中等

□ 低

□ 很低或完全没有

13、您对最近四周内全部性生活的满意程度如何？

□ 很满意

□ 满意

□ 一半满意，一半不满意

□ 不满意

□ 很不满意

14、您对最近四周内和配偶的性关系的满意程度如何？

□ 很满意

□ 满意

□ 一半满意，一半不满意

□ 不满意

□ 很不满意

15、您怎样评价最近四周内您对阴茎勃起和维持勃起的自信程度？

□ 很高 □ 低

□ 高 □ 很低

□ 中等

16、性生活时，您试图延长射精的困难程度如何？

□没有困难 □有些困难 □困难 □非常困难 □极度困难

17、射精是不是在你的意愿之前？

□几乎没有/没有（0%）

□少于一半次数（25%）

□约一半次数（50%）

□超过一半次数（75%）

□几乎都是/都是（100%）

18、在很小的刺激下射精？

□几乎没有/没有（0%）

□少于一半次数（25%）

□约一半次数（50%）

□超过一半次数（75%）

□几乎都是/都是（100%）

19、在你射精后（非自己意愿）有多少次感到焦虑、紧张等？

□从没有 □一些 □中等 □非常 □极度

20、对于你射精时间导致性伴侣不满足，你多关注？

□从没有 □一些 □中等 □非常 □极度

在过去的两周里, 你生活中以下症状出現的频率有多少？

1 感觉紧张，焦虑或急切

□没有 □有几天 □一半以上时间 □几乎天天

2 不能够停止或控制担忧

□没有 □有几天 □一半以上时间 □几乎天天

3 对各种各样的事情担忧过多

□没有 □有几天 □一半以上时间 □几乎天天

4 很难放松下来

□没有 □有几天 □一半以上时间 □几乎天天

5 由于不安而无法静坐

□没有 □有几天 □一半以上时间 □几乎天天

6 变得容易烦恼或急躁

□没有 □有几天 □一半以上时间 □几乎天天

7 感到似乎将有可怕的事情发生而害怕

□没有 □有几天 □一半以上时间 □几乎天天

1 做什么事都没兴趣, 沒意思

□没有 □有几天 □一半以上时间 □几乎天天

2 感到心情低落, 抑郁, 沒希望

□没有 □有几天 □一半以上时间 □几乎天天

3 入睡困难,总是醒着, 或睡得太多嗜睡

□没有 □有几天 □一半以上时间 □几乎天天

4 常感到很疲倦,沒劲

□没有 □有几天 □一半以上时间 □几乎天天

5 口味不好,或吃的太多

□没有 □有几天 □一半以上时间 □几乎天天

6 自己对自己不满, 觉得自己是个失败者,或让家人丟脸了

□没有 □有几天 □一半以上时间 □几乎天天

7 无法集中精力,即便是读报纸或看电视时,记忆力下降

□没有 □有几天 □一半以上时间 □几乎天天

8 行动或说话缓慢到引起人们的注意,或刚好相反, 坐臥不安,烦躁易怒易怒,到处走动

□没有 □有几天 □一半以上时间 □几乎天天

9 有不如一死了之的念头, 或想怎样伤害自己一下

□没有 □有几天 □一半以上时间 □几乎天天

请用7分制给下列问题打分，1(强烈反对)到7(强烈同意)

1. 我们的婚姻很好：（ ）分

2．我和配偶的关系很稳定： （ ）分

3．我们的婚姻很牢固： （ ）分

4．我和伴侣的关系让我很开心 ：（ ）分

5．我真的觉得和配偶是一个团队的一员： （ ）分

请用10分制给你和配偶的总体幸福水平打分， 1分(非常低)到10分(非常高)。

从各方面考虑，我们婚姻的幸福程度是： （ ）分
