# Supplementary material for: Sexual and psychological health of couples with azoospermia in the context of the COVID-19 pandemic
Source: PeerJ. 2021 Oct 20;9:e12162. doi: 10.7717/peerj.12162 (PMC8541304; doi:10.7717/peerj.12162)
Supplement: Supplemental Information 5 [file peerj-09-12162-s005.docx]

**性生活满意度调查问卷(女)**

**（此问卷是匿名及保密的，请您放心填写）**

**我们非常感谢及感激您对此项研究做出的贡献，祝您一切顺利，早日圆梦。**

年龄： 身高： cm 体重 ： kg

从事何种类型工作 □公务员 □专业技术人员 □企业管理人员 □职员

□工人 □农民 □个体户 □无 □其它：____________

个人年收入 □<5万元 □5-10万元 □10-15万元 □15-20万元 □＞20万元

要孩子几年了：_________ 年 娱乐方式：___________

是否服用药物： □是，何种药物：___________ □ 否

**以下题目请您选择一个您认为最符合您的情况的打对号**

教育程度 □高中及以下 □大专 □大学本科 □研究生及以上

饮食 □只吃素食 □以肉食为主 □素食肉食一半一半

工作及生活压力大吗 □非常大 □大 □一般 □不大 □没有

体育锻炼频率 □没有 □一个月2次及以下

□一周1次 □一周2次及以上

吸烟 □是，一天 支 □ 否

饮酒 □几乎每天 □常常 □有时 □很少 □从不

咖啡 □几乎每天 □常常 □有时 □很少 □从不

1.新型冠状病毒肺炎，是否给您带来焦虑？让您变得抑郁？

□是 □有一点 □ 否

2.在新型冠状病毒肺炎大流行期间，您的伴侣关系如何?

□很好 □一般 □恶化

3.在新型冠状病毒肺炎大流行期间，您的伴侣关系与之前相比如何?

□变好 □无变化 □变坏

4.在新型冠状病毒肺炎大流行期间，您的性欲与之前相比如何?

□增加 □不变 □减少

5.在新型冠状病毒肺炎大流行期间，您的性行为频率与之前相比如何?

□增加 □不变 □减少

6.在新型冠状病毒肺炎大流行期间，您的性生活满意度与之前相比如何?

□增加 □不变 □减少

7.在新型冠状病毒肺炎大流行期间，您在性活动之前或期间饮酒情况如何?

□增加 □不变 □减少

8.在新型冠状病毒肺炎流行期间，您的手淫频率是如何变化的?

□增加 □不变 □减少 □从来没有

9.在新型冠状病毒肺炎大流行期间，您们使用色情作品的频率有何变化?

□增加 □不变 □减少 □从来没有

10.在新型冠状病毒肺炎大流行期间，您的避孕套使用比例(在性接触中)有何变化?

□增加 □不变 □减少

11.新型冠状病毒肺炎，是否影响了您的年收入？

□增加 □不变 □减少

12.您是否因为新冠肺炎，想要推迟要孩子的时间

□是 □否

13.您是否因新冠肺炎的原因，在接受助孕治疗过程中遇到困难？

□是 □否

**如果遇到困难，哪方面的困难：**

14.在接受辅助生殖技术助孕过程中，进行新冠病毒核酸检测，是否增加了您的负担？

□是 □否

**每月**性生活次数： 次

15.性生活对您来说重要吗？

□非常重要 □重要 □一般 □不重要 □非常不重要

16.您是否有性交困难，性交障碍？

□从不 □极少 □很少 □有时 □常常 □几乎总是

17.性交时间

□<1分钟 □1-2 分钟 □3-4 分钟 □5-7分钟 □8-10 分钟

□11-15 分钟 □16-30 分钟 □>30 分钟

18.前戏时间

□<1分钟 □2-10分钟 □11-20分钟 □21-30分钟 □31-60分钟

□>60分钟

19.手淫获得高潮的可能

□几乎总是 □常常 □有时 □很少 □从不 □没有尝试过

20.非性交行为（如爱抚或者口交等）获得高潮的可能

□几乎总是 □常常 □有时 □很少 □从不 □没有尝试过

21.性交行为获得高潮的可能

□几乎总是 □ 常常 □有时 □很少 □从不 □没有尝试过

22. 近4周内，您感到有性欲望或对异性有性兴趣的频率如何?

□总是有或几乎总是 □大多数时候(超过一半的时间) □有时(大约一半的时间) □较少(不到一半的时间) □几乎没有或没有

23. 近4周内，您怎样评价您的性欲望或性兴趣的等级(或水平)？

□非常高 □高 □中等 □低 □很低或没有

24. 近4周内，在性行为或者性交时，您感受到性兴奋的频率如何？

□没有性行为 □总是能够 □大多数时候(超过一半的时间)

□有时(大约一半的时间) □较少(不到一半的时间) □几乎没有或没有

25. 近4周内，您在性行为或者性交时性兴奋的程度如何？

□没有性行为 □非常高 □高 □中等 □低 □很低或几乎没有

26. 近4周内，您在性行为或者性交时对性兴奋有足够的自信吗？

□没有性行为 □非常自信 □高度自信 □中度自信 □低度自信

□非常低或没有自信

27. 近4周内，您在性行为或者性交时有多少次对性兴奋感到满意？

□没有性行为 □总是或几乎总是 □大多数时候(超过一半的次数)

□有时(大约一半的次数) □较少(不到一半的次数) □几乎没有或没有

28. 近4周内，在性行为或性交时您经常感到阴道湿润吗?

□没有性行为 □总是或几乎总是 □大多数时候(超过一半的次数)

□有时(大约一半的次数) □较少(不到一半的次数) □几乎没有或没有

29. 近4周内，您在性行为或性交时阴道湿润的困难程度如何?

□没有性行为 □没有困难 □稍有困难 □困难 □非常困难

□极度困难或根本不能

30. 近4周内，在性行为或性交过程中，有多少时候您觉得能够保持阴道润滑(湿润)一直到性活动结束？

□没有性行为 □总是或几乎总是能 □大多数时候(超过一半的次数)

□有时(大约一半的次数) □较少(不到一半的次数) □几乎没有或没有

31. 近4周内，您维持阴道润滑(湿润)一直到性行为或性交结束的困难程度如何？

□没有性行为 □没有困难 □稍有困难 □困难 □非常困难

□极度困难或根本不能

32. 近4周内，当您受到性刺激或性交时，达到性高潮的频率有多少？

□没有性行为 □总是或几乎总是能达到 □大多数时候(超过一半的次数) □有时(大约一半的次数) □较少(不到一半的次数) □几乎不能或不能

33. 近4周内，您在性刺激或性交时，达到性高潮的困难程度如何？

□没有性行为 □没有困难 □稍有困难 □困难 □非常困难

□极度困难或根本不能

34. 近4周内，您对您在性行为或性交时达到性高潮的能力满意吗？

□没有性行为 □非常满意 □比较满意 □满意和不满各占一半

□不满意 □非常不满意

35. 近4周内，在性生活过程中您与丈夫的感情亲密度满意程度怎么样？

□没有性行为 □非常满意 □比较满意 □满意和不满各占一半

□不满意 □非常不满意

36.近4周内，您对您和丈夫(或性伴侣)的性关系满意吗？

□没有性行为 □非常满意 □比较满意 □满意和不满各占一半

□不满意 □非常不满意

37. 近4周内，您对您的性生活整体满意度如何？

□没有性行为 □非常满意 □比较满意 □满意和不满各占一半

□不满意 □非常不满意

38. 近4周内，在阴茎插入阴道**时**，有多少次您感到阴道不适或疼痛？

□没有尝试性交 □几乎没有或没有 □较少(不到一半的次数)

□有时(大约一半的次数) □大多数时候(超过一半的次数)

□总是或几乎总是

39. 近4周内，您在阴茎插入阴道**后**感觉阴道不适或疼痛的频率？

□没有尝试性交 □几乎没有或没有 □较少(不到一半的次数)

□有时(大约一半的次数) □大多数时候(超过一半的次数)

□总是或几乎总是

40. 近4周内，您在阴道插入过程中或结束后感到阴道不舒服或疼痛的程度如何？

□没有尝试性交 □非常低或没有 □低

□中度 □比较严重 □非常严重

在过去的两周里, 你生活中以下症状出現的频率有多少？

1 感觉紧张，焦虑或急切

□没有 □有几天 □一半以上时间 □几乎天天

2 不能够停止或控制担忧

□没有 □有几天 □一半以上时间 □几乎天天

3 对各种各样的事情担忧过多

□没有 □有几天 □一半以上时间 □几乎天天

4 很难放松下来

□没有 □有几天 □一半以上时间 □几乎天天

5 由于不安而无法静坐

□没有 □有几天 □一半以上时间 □几乎天天

6 变得容易烦恼或急躁

□没有 □有几天 □一半以上时间 □几乎天天

7 感到似乎将有可怕的事情发生而害怕

□没有 □有几天 □一半以上时间 □几乎天天

1 做什么事都没兴趣, 沒意思

□没有 □有几天 □一半以上时间 □几乎天天

2 感到心情低落, 抑郁, 沒希望

□没有 □有几天 □一半以上时间 □几乎天天

3 入睡困难,总是醒着, 或睡得太多嗜睡

□没有 □有几天 □一半以上时间 □几乎天天

4 常感到很疲倦,沒劲

□没有 □有几天 □一半以上时间 □几乎天天

5 口味不好,或吃的太多

□没有 □有几天 □一半以上时间 □几乎天天

6 自己对自己不满, 觉得自己是个失败者,或让家人丟脸了

□没有 □有几天 □一半以上时间 □几乎天天

7 无法集中精力,即便是读报纸或看电视时,记忆力下降

□没有 □有几天 □一半以上时间 □几乎天天

8 行动或说话缓慢到引起人们的注意,或刚好相反, 坐臥不安,烦躁易怒易怒,到处走动

□没有 □有几天 □一半以上时间 □几乎天天

9 有不如一死了之的念头, 或想怎样伤害自己一下

□没有 □有几天 □一半以上时间 □几乎天天

请用7分制给下列问题打分，1(强烈反对)到7(强烈同意)

1. 我们的婚姻很好：（ ）分

2．我和配偶的关系很稳定： （ ）分

3．我们的婚姻很牢固： （ ）分

4．我和伴侣的关系让我很开心 ：（ ）分

5．我真的觉得和配偶是一个团队的一员： （ ）分

请用10分制给你和配偶的总体幸福水平打分， 1分(非常低)到10分(非常高)。

从各方面考虑，我们婚姻的幸福程度是： （ ）分
